# Thymectomy for Morvan Syndrome Associated With Thymoma

**DOI:** 10.1016/j.atssr.2025.03.016

**Published:** 2025-04-03

**Authors:** Daichi Kakibuchi, Shunta Ishihara, Masanori Shimomura, Satoru Okada, Tatsuo Furuya, Masayoshi Inoue

**Affiliations:** 1 Division of Thoracic Surgery, Department of Surgery, Kyoto Prefectural University of Medicine, Kyoto, Japan; 2 Division of Thoracic Surgery, Japanese Red Cross Kyoto Daiichi Hospital, Kyoto, Japan

## Abstract

A 67-year-old man presented with lower back pain, numbness in the lower limbs, and general malaise. Over time, he experienced insomnia, abnormal behavior, muscle weakness, weight loss, orthostatic hypotension, and vesicorectal dysfunction. He was given a diagnosis of Morvan syndrome. Chest computed tomography revealed a 4.5-cm mass in the anterior mediastinum. He underwent steroid pulse therapy and therapeutic plasmapheresis, followed by robotic subxiphoid-optical extended thymectomy. His neurologic symptoms improved, and he was discharged 3 months after surgery. This case highlights the potential benefits of combining surgery with immunosuppressive therapy for managing Morvan syndrome and improving neurologic symptoms associated with thymoma.

Morvan syndrome is a rare autoimmune disorder characterized by peripheral nerve hyperexcitability, autonomic dysfunction, and central nervous system symptoms.^1^ It has been reported in association with thymoma in 37.9% to 56% of cases.^2^^,^^3^ However, Morvan syndrome occurs in fewer than 1% of patients with thymoma.^2^ We report a case of Morvan syndrome associated with thymoma in a patient who presented with severe neurologic symptoms.

A 67-year-old man presented with low back pain, numbness in the lower limbs, and general malaise. His symptoms gradually progressed, and after 1 month, he experienced insomnia and irritability. Subsequently, taste disturbances, loss of appetite, muscle weakness, and involuntary movements emerged, leading to daytime fatigue and difficulty writing. Three months after symptom onset, he consulted the general medicine department, where he was prescribed antidepressants. However, his symptoms did not improve, and he experienced hallucinations and urinary incontinence. He then visited a psychiatrist, who suspected dementia with Lewy bodies and initiated treatment. Despite this care, his condition continued to deteriorate, with subsequent orthostatic hypotension and dysphagia. After 11 months, he consulted a neurologist. Needle electromyography showed myotonic discharges (Figure 1A), and F-wave measurement in nerve conduction studies revealed repetitive discharges (Figure 1B), suggesting peripheral nerve hyperexcitability. Brain magnetic resonance imaging showed no specific abnormalities. Results of a test for serum anti–voltage-gated potassium channel complex antibodies were positive. He received a diagnosis of Morvan syndrome and was transferred to our hospital (Kyoto Prefectural University of Medicine, Kyoto, Japan). Chest computed tomography revealed a 4.5-cm mass in the anterior mediastinum, suspected to be thymoma (Figure 1C). Approximately 1 year elapsed from symptom onset to diagnosis.

**Figure 1:**
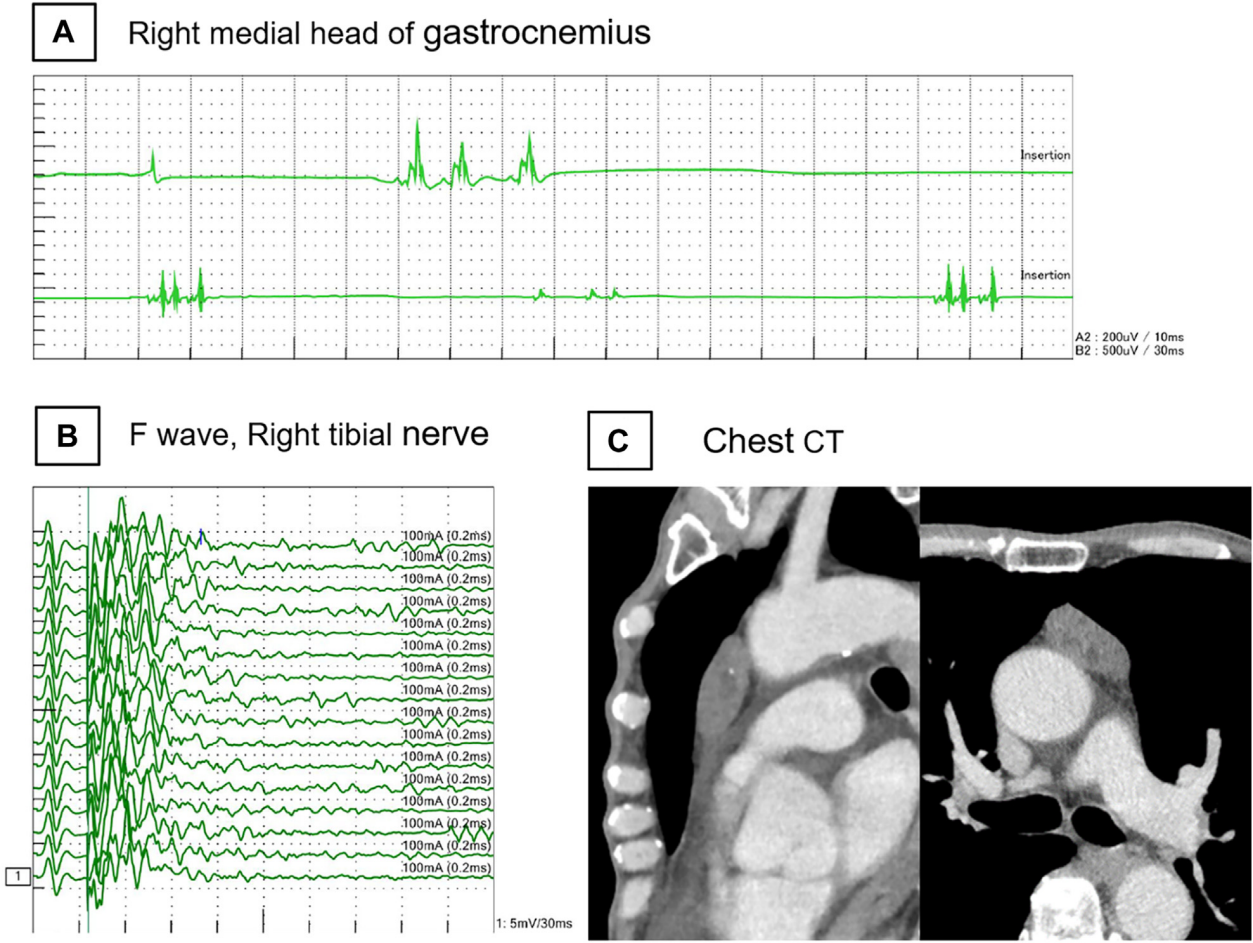
Preoperative clinical examination. (A) Needle electromyography showed myotonic discharge in the tested muscles of the limbs at rest, a finding that suggested overexcitation of peripheral nerves. (B) Repetitive discharge was observed in F-wave measurement in nerve conduction studies. (C) Chest computed tomography (CT) revealed a 4.5-cm mass in the anterior mediastinum.

On admission, he was bedridden, unable to communicate, and unable to tolerate oral intake. He was treated with steroid pulse therapy (1 g/d for 3 consecutive days) and therapeutic plasmapheresis (Figure 2A). After treatment, both central and peripheral nervous system symptoms improved. The prednisolone dosage was gradually tapered from 45 to 20 mg/d without symptom relapse. Fifty days after treatment initiation, he underwent robotic subxiphoid-optical thymectomy. The patient was in the supine position. The operating arms were placed in 3 ports, each 8 mm: the right sixth intercostal space (ICS) on the midclavicular line, subxiphoid (camera, 30 oblique view), and the left sixth ICS on the midclavicular line. An assistant port was placed at the right third ICS with an Air Seal port (Conmed).^4^ The tumor originated from the thymus and extended into the right thoracic cavity. An extended thymectomy was performed, removing both the tumor and the thymus gland up to the inferior pole of the thyroid gland. The patient’s postoperative course was uneventful, and his neurologic symptoms improved further. By postoperative day 4, he was able to take oral medication. He was transferred to a rehabilitation hospital on postoperative day 7. Histopathologic examination revealed spindle-shaped tumor cells (Figure 3A), whereas immunostaining showed cluster of differentiation 3–positive and terminal deoxynucleotidyl transferase–negative lymphocytes (Figures 3B, 3C). The final diagnosis was type A thymoma. Tumor classification was stage I according to the TNM classification and stage II according to the Masaoka staging system.

**Figure 2:**
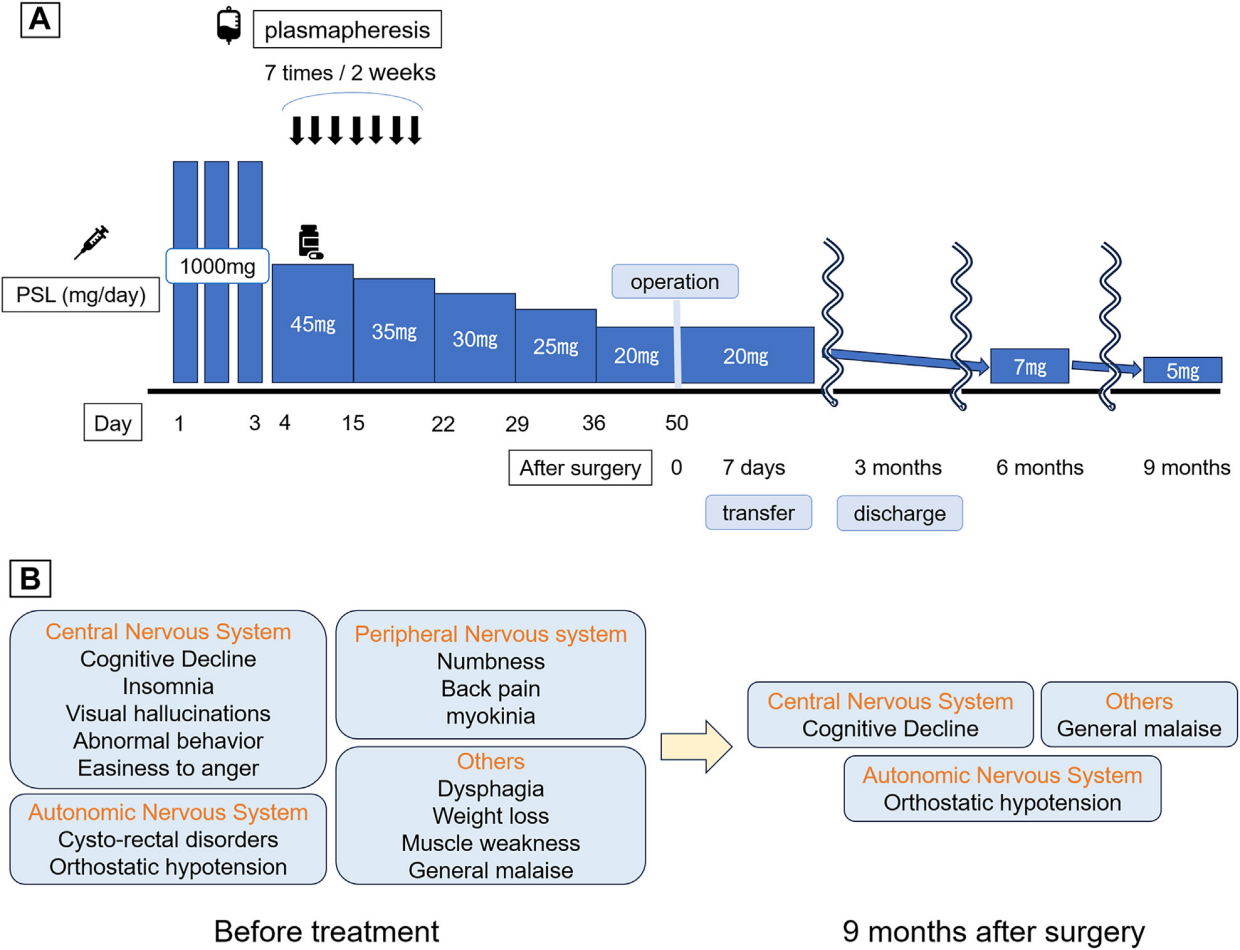
Clinical course. (A) Preoperative treatment and postoperative course. Surgery was performed after confirming no flare-up of symptoms. (B) Symptoms before treatment and residual symptoms 9 months postoperatively. (PSL, prednisolone.)

**Figure 3:**
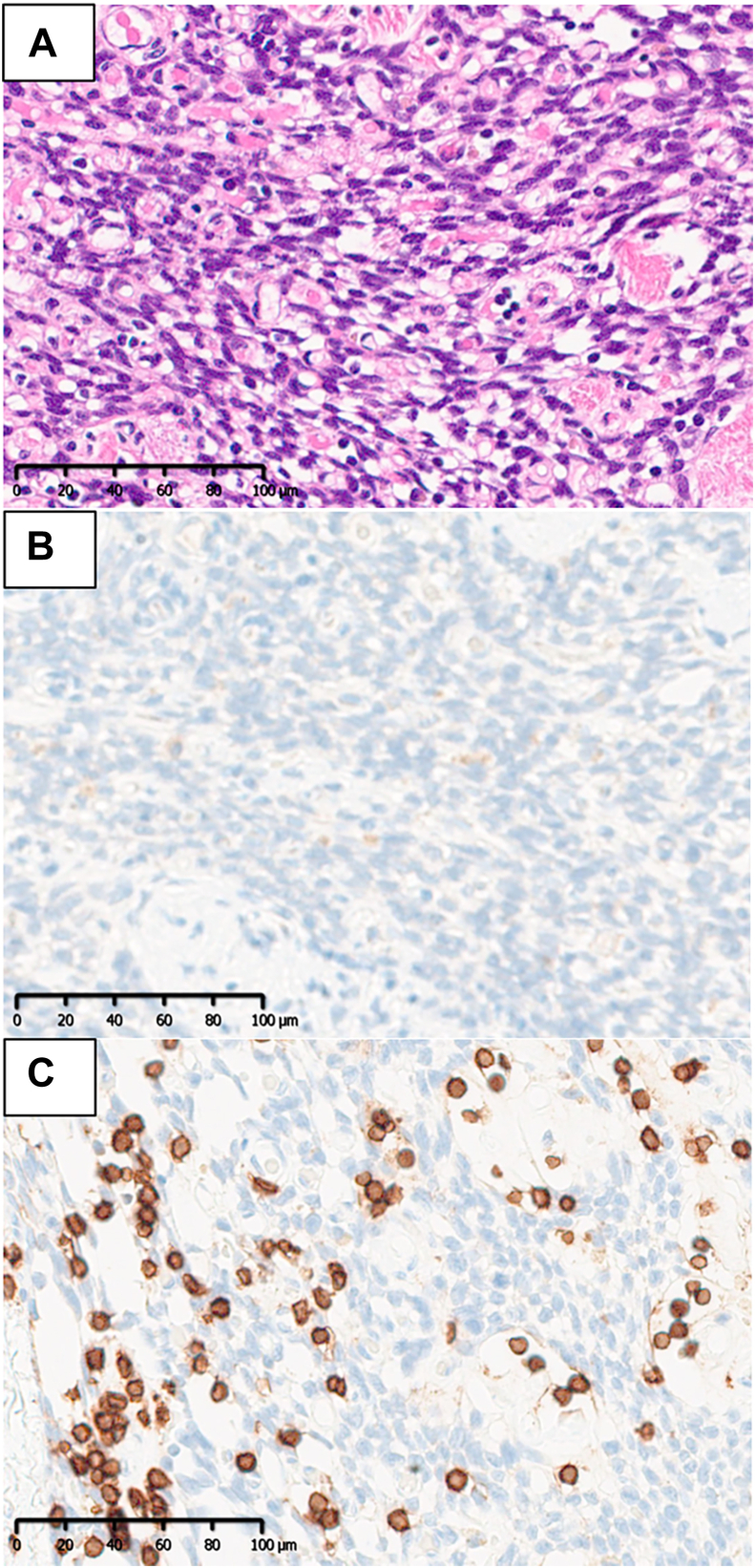
Microscopic pathologic findings. (A) Hematoxylin and eosin staining revealed atypical, weakly spindle-shaped cells arranged in a bundle-like arrangement, alternating with collagen fibers, and the nuclear fission pattern was inconspicuous. (Original magnification ×200.) (B) Immunohistochemical staining showed terminal deoxynucleotidyl transferase negativity. (Original magnification ×200.) (C) Immunohistochemical staining showed CD3 positivity. (Original magnification ×200.)

He was discharged home 3 months after surgery with further improvement in neurologic symptoms. By 6 months, he was ambulatory, and by 9 months, his prednisolone dosage was reduced to 5 mg/d. At 22 months postoperatively, there was no recurrence of thymoma (Figure 2B), and neurologic symptoms, including orthostatic hypotension, cognitive decline, and general malaise, remained stable.

## Comment

Morvan syndrome is an extremely rare neurologic disorder, with a prevalence of fewer than 1 in a million.^1^ It typically manifests as pain, dysesthesia, myokymia, autonomic dysfunction (eg, hyperhidrosis, orthostatic hypotension), and central nervous system symptoms such as insomnia, hallucinations, and disorientation.^1^, ^2^, ^3^ In thymoma-associated cases, Morvan syndrome frequently coexists with other autoimmune diseases, such as myasthenia gravis.^2^^,^^3^ Therefore, when patients with thymoma present with central or peripheral neurologic symptoms, Morvan syndrome should be considered.

Robotic-assisted thymectomy reduces postoperative complications and allows for faster recovery compared with the transsternal approach.^5^ In addition, the robotic subxiphoid approach provides comfortable access to bilateral phrenic nerves,^4^ and we averted postoperative complications, including phrenic nerve injury. In patients with severe neurologic dysfunction, early mobilization is crucial, and the robotic subxiphoid approach may help optimize perioperative management to avoid postoperative complications.

Given its nonspecific symptoms, such as insomnia and fatigue, Morvan syndrome is often misdiagnosed as dementia or depression. This multidimensional clinical presentation underscores the importance of early clinical suspicion. Electromyographic findings, including fasciculations, multiplets, and after-discharges, aid in diagnosis. Conversely, magnetic resonance imaging often performed in cases of nervous system hyperexcitability, typically yields nonspecific results, thus contributing to diagnostic delays.^1^ Moreover, delays frequently occur between symptom onset and diagnosis with treatment initiation, thus highlighting the need for heightened clinical awareness. In the present case, 1 year elapsed before diagnosis and treatment initiation. Despite effective treatment, some symptoms persisted, necessitating oral steroid administration (5 mg/d). To prevent prolonged illness, Morvan syndrome should be considered in cases of thymoma in patients presenting with nonspecific hyperexcitability of the central and peripheral nervous systems.

Pharmacotherapy is the mainstay of Morvan syndrome treatment. Previous reports suggest that plasmapheresis is the most effective therapy,^1^, ^2^, ^3^ often combined with corticosteroids or immunosuppressive agents.^2^, ^3^, ^4^^,^^6^ In cases involving thymoma, surgical intervention is required.^2^^,^^3^^,^^6^ Abou-Zeid and colleagues^2^ reported that 50% of Morvan syndrome cases with thymic abnormalities showed lasting improvement after thymectomy, whereas most of the remaining cases were controlled with additional immunosuppressive therapy. In the present case, a combination of pharmacotherapy and thymectomy led to significant neurologic improvement.

Interestingly, some reports describe Morvan syndrome developing after thymectomy or chemotherapy for thymoma, a finding suggesting that thymectomy itself may act as a trigger.^7^ However, further research is needed to clarify this phenomenon.

In conclusion, Morvan syndrome associated with thymoma can be effectively managed with a combination of extended thymectomy and immunosuppressive therapy. Early recognition and prompt intervention are crucial for preventing prolonged neurologic impairment.
